# Knee focal cartilage defect location heat map and local surface morphology characterisation: Insights for focal knee resurfacing implant design

**DOI:** 10.1002/jeo2.70216

**Published:** 2025-04-01

**Authors:** Majid Mohammad Sadeghi, Erkan Aşık, Pieter Emans, Frank Zijta, Nazli Tümer, Gabrielle Tuijthof, Alex Roth

**Affiliations:** ^1^ Department of Orthopedic Surgery, Joint Preservation Clinic Maastricht University Medical Center Maastricht The Netherlands; ^2^ Department of Orthopedic Surgery, Research School CAPHRI Maastricht University Maastricht The Netherlands; ^3^ Avalanche Medical BV Maastricht The Netherlands; ^4^ Department of Radiology Maastricht University Medical Center Maastricht The Netherlands; ^5^ Department of Biomechanical Engineering Delft University of Technology Delft The Netherlands; ^6^ Department of Biomechanical Engineering University of Twente Enschede The Netherlands

**Keywords:** cartilage defect location heat map, cartilage surface morphology, knee focal cartilage defects, lateral femoral condyle, medial femoral condyle, statistical shape modelling, trochlea

## Abstract

**Purpose:**

The objective of this work was to characterise the dimensions of knee focal cartilage defects, generate a heat map of cartilage defect locations, and characterise the native articular surface morphology at the most common defect sites to aid in the design of generic focal knee resurfacing implants (FKRIs).

**Methods:**

Healthy femoral cartilage was segmented from knee magnetic resonance imaging scans of 70 patients eligible for FKRI surgery. Cartilage defects were manually reconstructed to create three‐dimensional (3D) native cartilage and cartilage defect models. A 3D statistical shape model of the native cartilage was created and 200 artificial models with varying shapes were generated. A heat map showing the frequency of defect occurrence location was created, and the size and aspect ratio of the defects were determined. Local radii of curvature were calculated at five locations that exhibit high defect occurrence for the medial and lateral condyles and in the trochlear groove.

**Results:**

The median defect size and aspect ratio were 2.7 and 1.6, respectively. Cartilage defect location frequency heat maps were successfully generated for the medial and lateral condyles. The median mediolateral and anteroposterior radius of curvature at the medial condyle hotspot were 17.5 mm and 40.8 mm respectively, and 22.6 mm and 33.8 mm at the lateral condyle hotspot.

**Conclusions:**

Characteristics of knee focal cartilage defect properties such as the size, their spatial distribution and local articular cartilage surface morphology offer valuable insights for the design of generic FKRIs.

**Level of Evidence:**

Level IV.

**Abbreviations**
3D: three‐dimensional
CT: computed tomography
FKRI: focal knee resurfacing implant
GPA: generalised procrustes alignment
ICP: iterative closest point
LFC: lateral femoral condyle
MFC: medial femoral condyle
MRI: magnetic resonance imaging
PCA: principle component analysis
PSI: patient‐specific implants
ROC: radius of curvature
SSM: statistical shape model
TKR: total knee replacement
WATS: water‐selective gradient‐echo
WORMS: Whole‐Organ Magnetic Resonance Imaging Score

## INTRODUCTION

Focal cartilage defects in the knee can result in pain, reduced quality of life, impaired physical activity, and can lead to the development of osteoarthritis if left untreated [9, 24]. Several biological cartilage repair techniques can be employed for the treatment of focal cartilage defects, including microfracture, autologous chondrocyte implantation, osteochondral autograft transfer, and osteochondral allograft transplantation (OCA) [50]. Focal knee resurfacing implants (FKRIs) are an emerging class of permanent implants typically intended for the middle‐aged population (40–60 years) [4]. FKRIs are considered when biological cartilage repair procedures and total knee replacement are not indicated due to either impaired regenerative capacity or concerns related to implant longevity [7, 40, 51]. Biological cartilage repair treatments tend to show inferior clinical outcomes in middle‐aged patients in comparison to younger patients, whereas FKRIs do not show these age‐related drawbacks [28].

The objective of FKRIs is to restore the knee's articulating surface to its native morphology using a permanent implant [27], thereby restoring the load‐bearing capacity of the affected compartment. While patient‐specific implants (PSI) offer advantages in terms of customised fit, they pose challenges related to the need for patient‐specific planning and logistics, and there is insufficient evidence to determine if PSIs translate into improved outcomes [37]. Finite element analysis studies suggest that FKRI material mechanical properties critically effect peak contact pressures in opposing native tissues [5, 31], with critically high peak contact pressures in turn causing potential deleterious effects on cartilage quality [1]. In contrast to the use of metallic implants, the use of more biomimetic materials in FKRIs could offer an alternative strategy for FKRI improvement without having to utilise a patient‐specific approach [56, 63]. To design and optimise generic FKRIs, a complete understanding of the characteristics of the defect site across the population is necessary, which includes the size‐, shape‐, and location distribution, along with the morphology of the original cartilage surface at the defect locations.

Current literature that describes knee cartilage defect characteristics in detail is limited. The defect locations are typically classified into general anatomical sections [11, 34, 60, 61] (i.e., patella, trochlea, medial/lateral femoral condyles and medial/lateral tibial plateaus). These sections are rarely divided into smaller subsections for better accuracy [6]. While this level of detail may suffice for descriptive purposes, detailed quantitative data is needed when it comes to implant design. For instance, the variation of cartilage surface morphology over different locations has critical implications for implant surface shape design.

The morphology of the femoral condyle has been topic of previous research, primarily focused on characterisation of the overall shape for total knee replacement implant design purposes. Different studies have tried to estimate the general curvatures of the femoral condyle surface and the trochlea [10, 26, 32, 43, 59]. However, when it comes to focal cartilage defect treatments, it becomes crucial to account for the local morphology of the cartilage surface. For instance, studies have demonstrated that a mismatch in local curvature during OCA treatments can lead to negative midterm clinical outcomes [22].

Statistical shape modelling (SSM) is a technique that analyzes a set of geometrical objects and identifies their shape variations, for instance joint morphology, across a population sample set [23, 52]. SSMs are commonly used to design and optimise population‐based generic implants, such as total knee replacements [15]. Magnetic resonance imaging (MRI) based SSM data can also be used to supplement computed tomography (CT) scan data of specific patients, thereby assisting the design of CT‐based patient‐specific total knee arthroplasty implants to decrease input image acquisition time without compromising design accuracy [55].

The objective of this work is to characterise knee focal cartilage defect properties including size, shape, and location distribution, and characterise the local articular cartilage surface morphology at sites where cartilage defects commonly occur using an SSM. While this study is explorative in nature, our hypothesis is that the most common areas where cartilage defects occur generally coincide with the areas that experience the highest loads during normal gait.

## MATERIALS AND METHODS

MRI scans of patients eligible for FKRI treatment were obtained and healthy femoral cartilage regions and reconstructed defect areas were segmented separately. The size and aspect ratio (ratio of maximum length to width) of the cartilage defects was determined. A colour‐mapping scheme (heat map) was used to visualise the spatial distribution of cartilage defects over the femoral cartilage surface to identify regions with the greatest defect incidence. A three‐dimensional (3D) SSM of the distal femoral cartilage surface was generated using the segmented reconstructed cartilage models. Using the 3D SSM, a set of 200 artificial cartilage models with varying shapes was generated. Finally, the local surface radii of curvatures of articular cartilage surface was measured at the specific high‐incidence regions for the set of artificial cartilage models created.

### Cohort selection

A retrospective evaluation was conducted on knee MRI scans of 115 patients who were considered for FKRI surgery due to a full‐thickness femoral focal cartilage defect > 1 cm^2^ in patients with a body mass index < 30 and a ligamentous stable knee. Patients with arthritic joint conditions (maximum grade 1 Outerbridge classification for cartilage outside the defect area), kissing lesions, more than 50% volume loss of the meniscus in the same compartment, and those with Whole‐Organ Magnetic Resonance Imaging Score (WORMS) [46] of 2 and 3 (indicating high subchondral femoral condyle bone attrition) were excluded from the study [4]. The cutoff values of 2 in the WORMS score was selected to ensure that the degree of bone attrition of the articular cartilage surface falls within a range where the conditions of the joint remain a suitable indication for FKRI treatment. Varus/valgus misalignment was not an exclusion criterion, as patients with >5° varus/valgus misalignment typically undergo a concomitant high tibial osteotomy for correction of the leg axis. After exclusion of cases, a final set consisted of the MRIs of 70 patients (42 males, 28 females, age: 43±10). All the MRIs were acquired using a three‐dimensional water‐selective gradient‐echo (3D WATS) sequence. The voxel size was 0.5 × 0.5 × 1 mm in axial, coronal, and sagittal planes respectively. Medical ethical approval for this study was received from the committee of Maastricht University Medical Center (MUMC) with reference number METC 2023‐3691.

### Tissue segmentation

Joint tissues including the articular cartilage and bone were automatically identified and segmented using MR ChondralHealth 3.1.0 (Siemens Healthineers AG, Forchheim, Germany) [29]. Manual corrections and refinements of the segmentations were performed afterwards, using 3D Slicer software 5.0.3 [30]. Manual correction of the automatic segmentations was performed by an engineer (MS) and an expert musculoskeletal radiologist (FZ) with 16 years of experience verified all the segmented image sets by reviewing the MRI slices and evaluating the correct labelling of the tissues. Initially, only the healthy cartilage regions were segmented and saved as a separate model, excluding the damaged cartilage area (focal cartilage defect). Healthy and injured cartilage were defined based on the ICRS Articular Cartilage Injury Classification System. Healthy tissue was defined to correspond with ICRS Grade 0 and I tissue, and the damaged cartilage defect area was defined to correspond with ICRS Grade II, III, IV tissue.

Next the damaged cartilage areas were manually reconstructed, following the contours of the subchondral bone plate and the adjacent cartilage. The resulting reconstructed “native” cartilage segmentation was saved as a new model. Through a Boolean subtraction operation between the native cartilage model and the model containing only healthy cartilage regions, a segmentation of the focal cartilage defect was attained, and the defect model was saved separately. Segmented models were used to obtain three‐dimensional triangulated surface models using 3D Slicer software. To ensure that all the knee models were oriented the same way, right‐sided knee models were mirrored in the sagittal plane.

### Defect geometrical characteristics

To measure the size of a defect, the articulating surface area of the defect model was calculated. To define the major axis of the defect, the pairwise distances between all points on the surface of the defect model were calculated, and a line was constructed by connecting the two points that exhibited the greatest distance. The minor axis of the defect was defined as the line connecting the most distanced points on the defect model in the direction perpendicular to the major axis. The aspect ratio of the defect was then calculated as the ratio between the length of the major axis and the length of the minor axis.

### Statistical shape model

A 3D SSM of the femoral cartilage was created in Matlab (Matlab R2022a, The Mathworks, Inc., Natick, MA) using a similar approach as described by Audenaert et al. [3]. An iterative closest point (ICP) based non‐rigid registration algorithm was used to establish corresponding vertices across the 70 cartilage surface models, by randomly selecting a cartilage model as a reference [3]. Generalised Procrustes Alignment (GPA) [18] was used to remove differences in position and orientation that do not contribute to the variation in the geometry. The cartilage models were not scaled to reflect size differences in the data, which is considered to be important geometrical information to retain in this study. Principle component analysis (PCA) was performed on the covariance matrix of the corresponding vertices of the cartilage models to find the principle modes of variations and their corresponding variances. Using the SSM, a new cartilage instance can be expressed as:

(1) $$S=\bar{S}+\text{Pb},$$

where S is a vector containing the vertices of the cartilage model represented by the SSM, $\overset{®}{S}$ is the mean cartilage shape, P is the matrix consisting of the modes of shape variation, and b is a vector of weights or coefficients, where each element corresponds to a particular mode of variation [52]. Varying the entries of the vector b in Equation (1) by sampling them from a Gaussian distribution, a set of 200 cartilage models was generated. The distribution of anteroposterior length versus the mediolateral width of the samples was graphically depicted and compared to the input data to determine if the resulting anatomical variability of the created samples is sufficiently representative of the original input set [15]. The anteroposterior length was defined as the distance between the most anterior cortex point and the line connecting the most posterior point of medial condyle and the most posterior point of lateral condyle. The mediolateral width was defined as the distance between the most lateral point of lateral condyle and the most medial point of medial condyle as described in [47].

### Defect location frequency heat map

**Figure 1:**
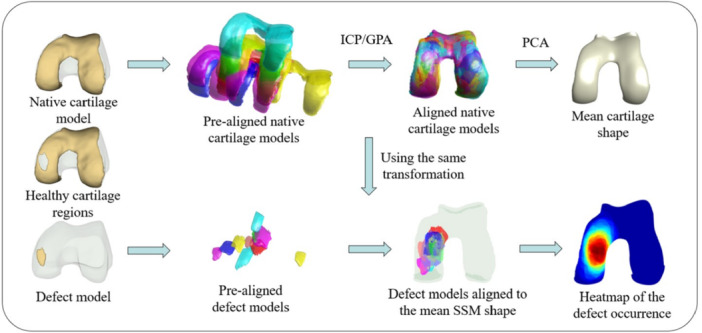
The workflow for generating the Statistical Shape Model (SSM) and the heat map are schematically depicted. Native cartilage models are obtained after segmentation of healthy cartilage regions and reconstructed cartilage defects sites based on MRI scans obtained from patients eligible for FKRI surgery. Iterative closest point (ICP) and generalised procrustes analysis (GPA) algorithms are used for registration and alignment of native cartilage models. Principal component analysis (PCA) is then employed to construct the SSM. The transformation matrices that were used to align the native cartilage models are applied to align the defect models. The defect location frequency heat map is generated by colour‐coding the mean cartilage shape's surface based on the number of overlapping defects at each point.

The 70 reconstructed “native” cartilage models were scaled and aligned to the mean SSM shape using GPA. The transformation matrices obtained from this alignment for each cartilage model were then utilised to align the corresponding defect models with the mean cartilage shape. Next, for each point on the surface of the mean SSM shape, the number of overlapping defect models was counted. Using this method, the frequency of cartilage defect occurrence at each location on the cartilage surface was extracted. By colour‐coding the surface of the mean cartilage shape based on the number of overlapping defects at each point, a heat map was created that shows the frequency of damage occurring at different locations. Figure 1 presents the workflow for generating the SSM and the heat map of the defect location frequency.

### Characterising cartilage surface morphology

**Figure 2:**
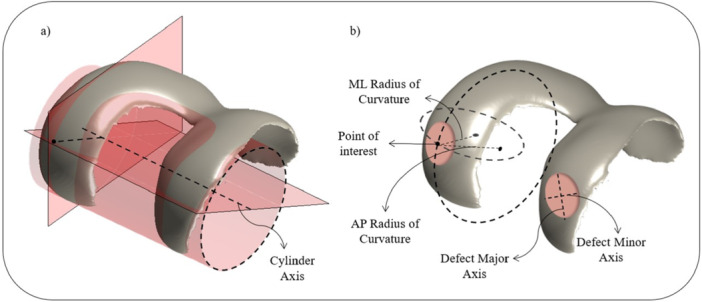
A cylinder is fitted to the cartilage surface and its axis is employed to define the planes for measuring the anteroposterior and mediolateral radii of curvature (a). The major and minor axes of cartilage defects are illustrated, along with the locally measured anteroposterior and mediolateral radii of curvature within a circular elliptical region centred on a point of interest on the cartilage surface (b).

To evaluate the local morphology of the cartilage surface at an arbitrary point of interest, first the articulating surface of the posterior condyles was selected and then a cylinder was fitted to it using a least square method as described in [49]. The cylinder's axis was used to determine the orientation of the planes utilised for measuring the curvatures. The first plane had the cylinder axis as its normal vector and intersected the cartilage surface at the selected point of interest, enabling the measurement of the anteroposterior radius of curvature (ROC). The second plane passed through the selected point of interest on the cartilage surface while encompassing the cylinder axis, facilitating the measurement of the mediolateral ROC. Once the two planes were determined for the selected point of interest on the cartilage surface, a section of the articulating surface was isolated from the remainder of the cartilage model. This section had an elliptical shape with the point of interest at its center and had a predefined surface area chosen to match the median size of the defects. The ratio of the major axis over the minor axis of the elliptical section was chosen to represent the median aspect ratio of the defects. The orientation of major axis of the section was chosen to be in the anteroposterior direction based on the orientation of the major axis of the majority of the defects. A circle was fitted [48] to the isolated surface in each of the planes defined. The radii of these circles are the radii of curvatures of the cartilage surface at the point of interest with the chosen surface area (Figure 2).

The radii of curvature were determined at five points on the medial femoral condyle (MFC) and lateral femoral condyle (LFC). These points were chosen over an area with a high frequency of defect occurrence and were equally spaced along the anteroposterior direction. Insufficient data was gathered for the trochlea to present a proper heat map, hence five points that represent frequent cartilage defect locations based on a surgeon's experience (PE) were chosen on the trochlear groove. Curvature values were also determined at these points. The measurements were performed for the selected points for all 200 artificially generated cartilage samples to evaluate the variation of curvatures within the target population.

### Data analysis

Descriptive statistics were used to describe cartilage defect size and aspect ratio for all defects and the MFC and LFC separately. A heat map was generated to visually depict the spatial distribution of cartilage defect locations on the MFC and LFC separately. Anteroposterior and mediolateral ROC values were quantified for the five points of interest on the MFC, LFC and trochlea and presented using descriptive statistics. To verify the quality of the created SSM, the compactness, accuracy, and generalisation ability of the model were assessed, as described by Audenaert et al. [2]. The compactness of the SSM model was determined by calculating the cumulative variance of the modes. The accuracy of the SSM model was assessed by computing the root mean square distance between the vertices of a training shape and its reconstructed version using Equation (1). This was analysed for different numbers of modes. The minimum number of modes to be kept was found when the accuracy level dropped below the voxel size of the training MRI scans for the first time. To evaluate the generalisation ability of the model, the accuracy of reconstructing cartilage instances that were not included in the training data was determined using varying numbers of principle modes of variation.

## RESULTS

**Figure 3:**
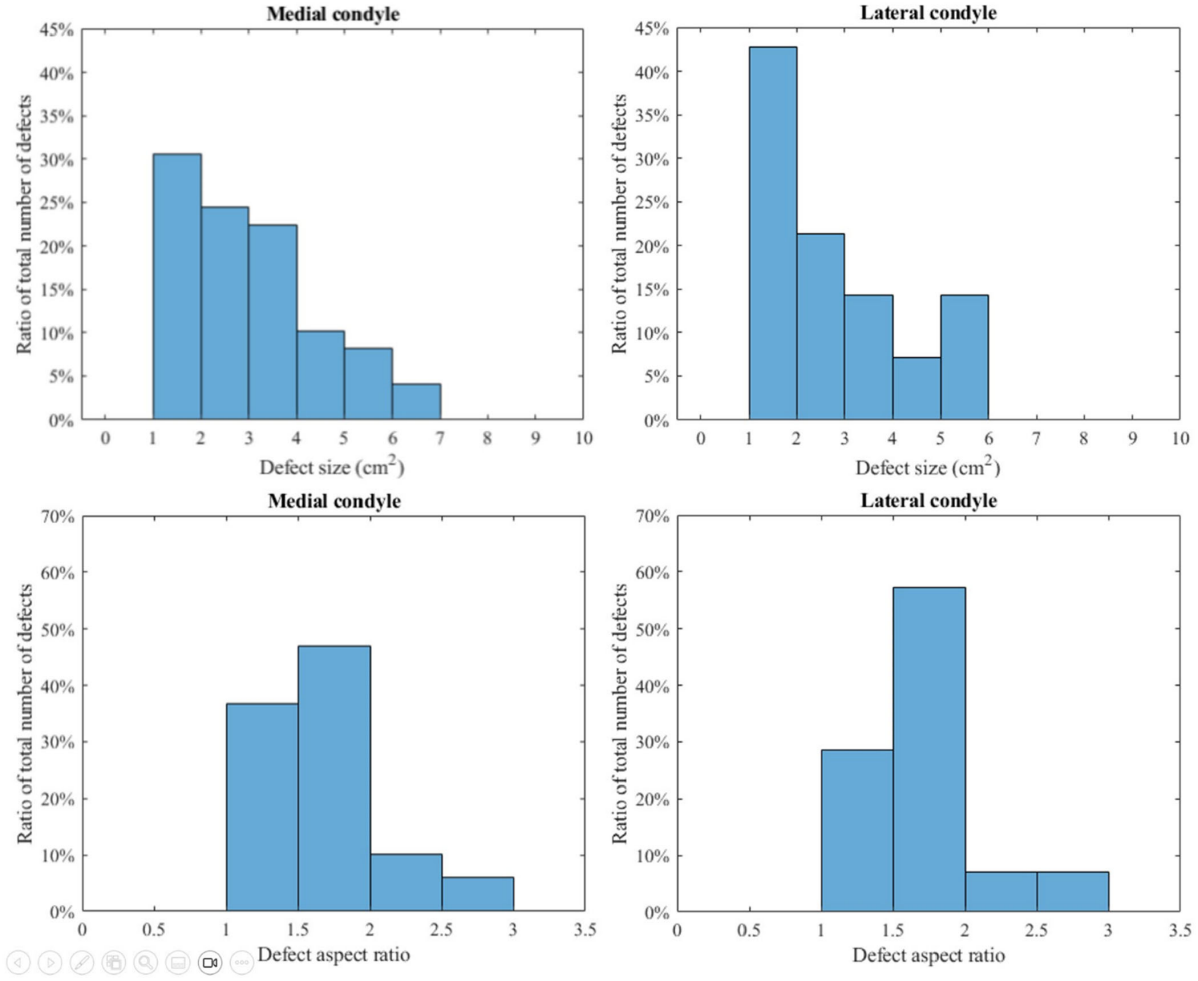
Distribution of defect size and aspect ratio for the medial condyle containing 49 defects (left) and lateral condyle containing 14 defects (right).

**Figure 4:**
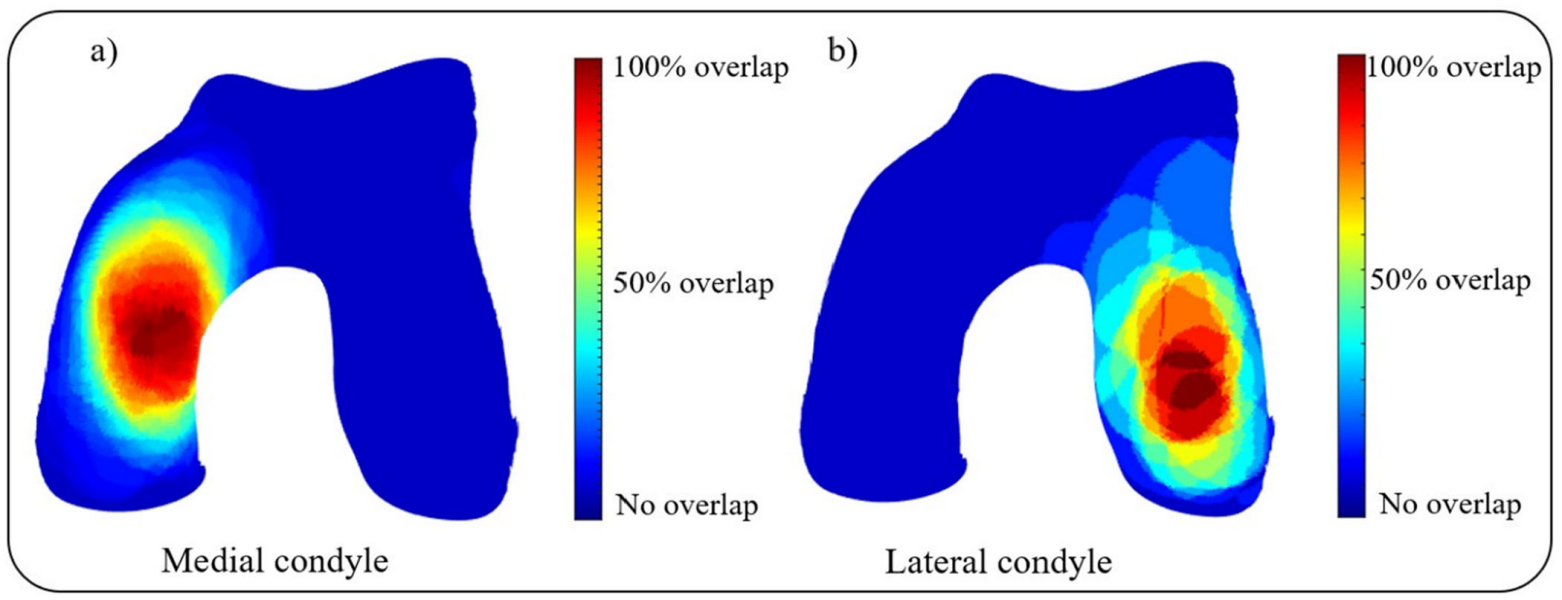
The (osteo)chondral defect location heat maps for the medial (a) and lateral (b) condyles.

The sizes and aspect ratios of the cartilage defects included in the current study are described in Table 1 and Figure 3. Two heat maps showing the frequency of cartilage defect occurrence location were created for the medial (49 defects) and lateral (14 defects) condyles, revealing the locations on the cartilage surface with the highest defect occurrence (Figure 4). Insufficient patient data was available to generate a meaningful heat map for trochlear defects (seven defects).

**Table 1:** The sizes and aspect ratios of the defects collectively and separately for medial and lateral condyle defects.

Location	Size			Aspect ratio		
	Median (cm^2^)	Interquartile range (cm^2^)	Range (cm^2^)	Median	Interquartile range	Range
Medial and lateral defects	2.7	1.7–4.0	1–6.6	1.6	1.4–1.9	1.1–3
Medial defects	2.8	1.7–3.9	1–6.6	1.7	1.4–1.9	1.2–2.8
Lateral defects	2.0	1.5–4.0	1.2–5.9	1.6	1.4–1.7	1.1–3

**Figure 5:**
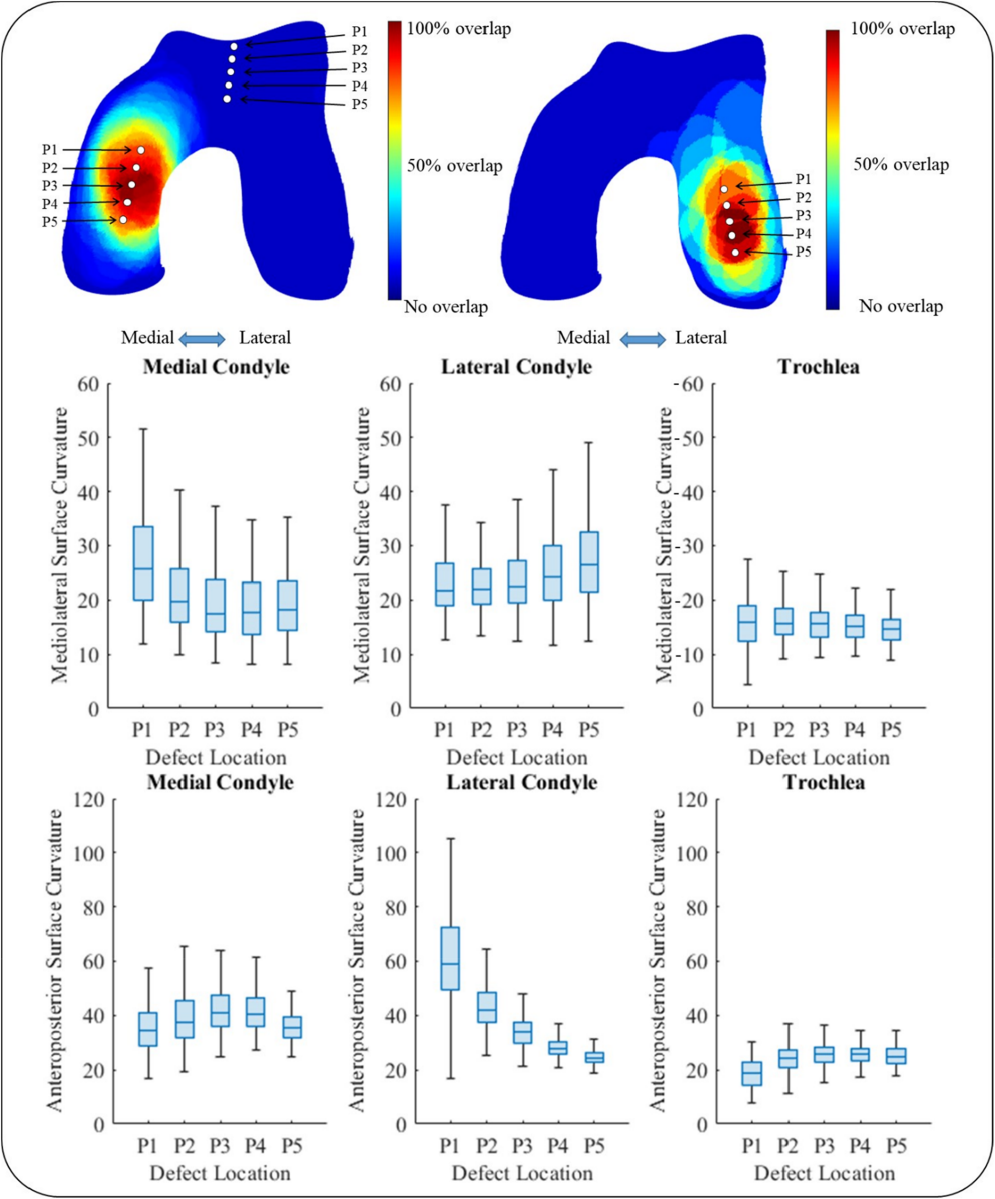
Mediolateral and anteroposterior radii of curvatures for five points selected on the femoral condyles and trochlea of 200 cartilage models generated using the developed Statistical Shape Model (SSM). Points 1–5 are chosen over an area with a high frequency of defect occurrence for medial and lateral condyles, with Point 3 placed on the highest defect occurrence location. The box plots display the distribution of curvature values for each point, presenting the median value by the line inside of the box, the lower and upper quartiles by the top and bottom edges of each box, and the minimum and maximum values by the whiskers that extend above and below each box.

A SSM of the distal femoral cartilage was successfully generated and local ROCs were measured. The five points selected on each of the three regions (the medial condyle, lateral condyle, and trochlea) and their corresponding curvature values are presented in Figure 5. Point 3 was placed on the highest defect occurrence location on the medial condyle. On the lateral condyle, Point 3 was placed in a central location of the high defect occurrence area. An ellipse with a surface area of 2.7 cm^2^ (median surface area of all the defects), and an aspect ratio of 1.6 (median aspect ratio of all the defects), translating into an ellipse with major and minor axes lengths of 24.2 mm and 14.2 mm, respectively, was used as region of interest for local ROC determination. Positive values present convex surface curvatures and negative values present concave surface curvatures. To give an example, the median mediolateral radius of Point 3 was 17.5 mm on the medial condyle, 22.6 mm on the lateral condyle and −15.7 mm on the trochlear groove. The median anteroposterior radius of Point 3 was 40.8 mm on the medial condyle, 33.8 mm on the lateral condyle, and 25.6 mm on the trochlear groove.

**Figure 6:**
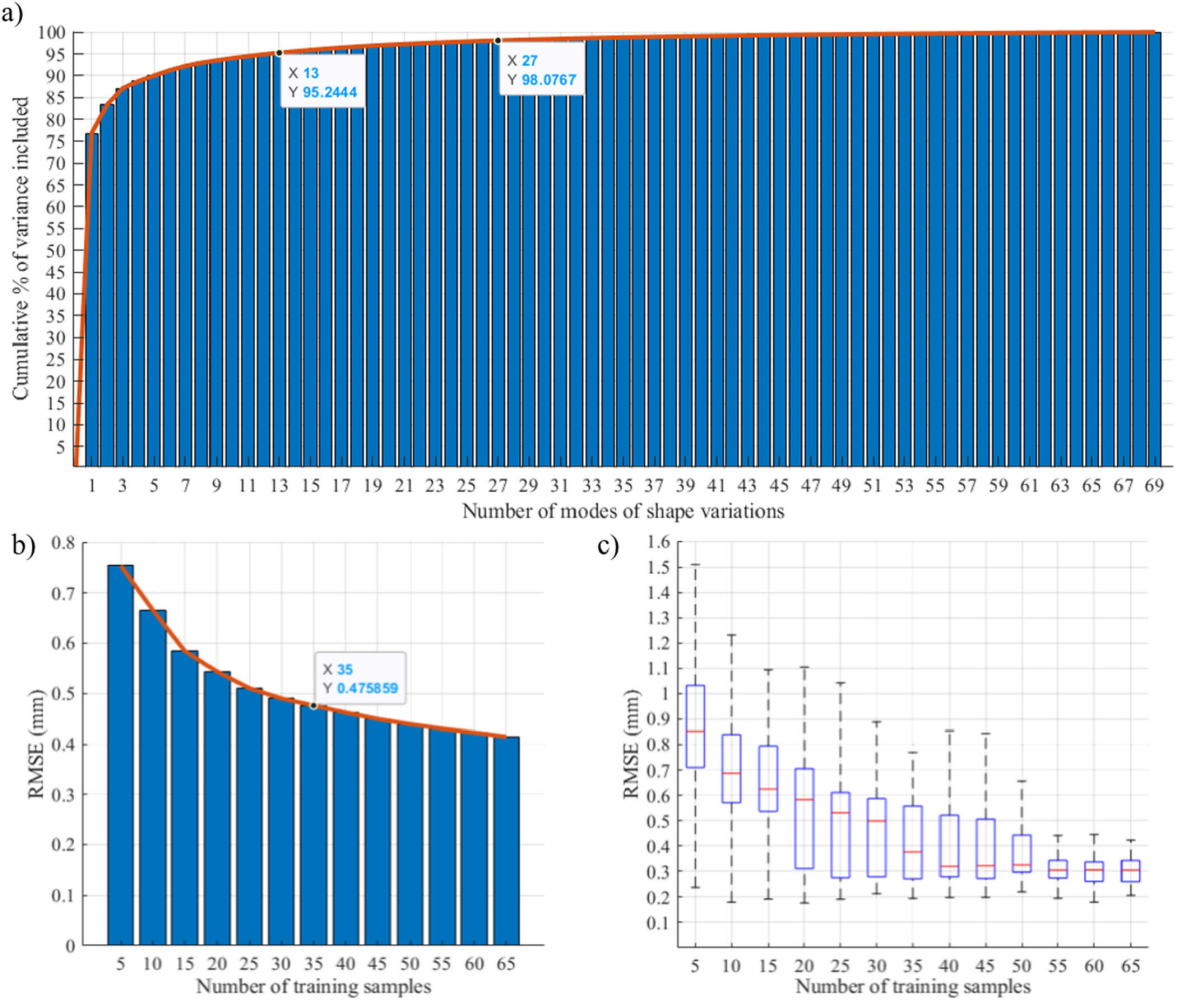
Results of the Statistical Shape Model (SSM) compactness, accuracy, and generalisation ability evaluation. (a) SSM compactness is presented as the cumulative variation of data included in the model based on the number of shape modes. The first 13 and 27 modes of shape variation account for 95% and 98% of the cartilage shape variation within the studied population, respectively. (b) Accuracy of the SSM based on the number of training samples; using the first 35 modes of variation, the accuracy of the SSM reached a value (0.48 mm) below the resolution of the magnetic resonance imaging (MRI) images (0.5 mm) for the first time, and (c) generalisation of the SSM based on the number of training samples. The generalisation ability of the SSM reached a value (0.30 mm) below the resolution of the MRI images (0.5 mm) for the first time when the first 55 modes of shape variation were used.

The results of SSM evaluation are presented in Figure 6. The first 13 and 27 modes of shape variation account for 95% and 98% of the cartilage shape variation within the studied population, respectively. Using the first 35 modes of variation, the accuracy of the SSM reached a value (0.48 mm) below the resolution of the MRI images (0.5 mm) for the first time. The generalisation ability of the SSM reached a value (0.30 mm) below the resolution of the MRI images for the first time when the first 55 modes of shape variation were used. As a result, at least the first 55 modes of variation must be used to generate artificial models using the SSM with accuracy below the resolution of the MRI images. In this study, all modes of variation were used for generating the sample set of 200 models for morphological analysis.

**Figure 7:**
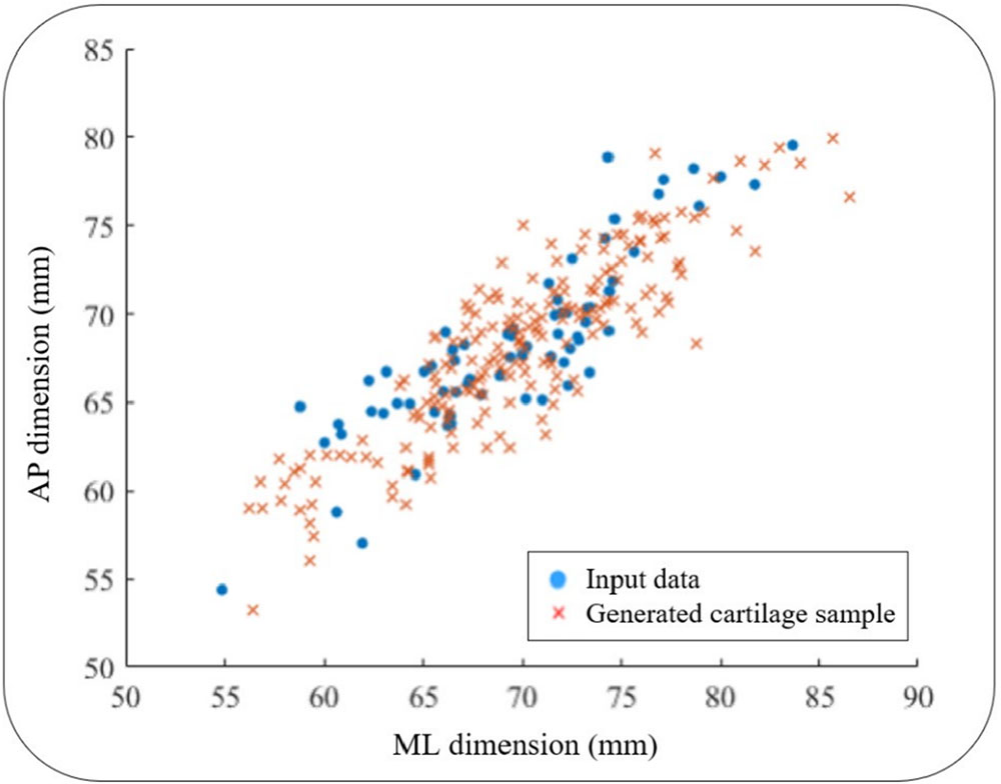
Distribution of the anteroposterior and mediolateral dimensions of the cartilage in the input data and the 200 generated cartilage samples. AP, anteroposterior, ML, mediolateral.

The distribution of anteroposterior length versus the mediolateral width for the data set of 200 artificially generated samples showed a significant overlap with that of the input data, which shows that the anatomical variation in the generated cartilage samples closely match the input data (Figure 7).

## DISCUSSION

This study presents heat maps that visualise the spatial distribution of cartilage defects in the knee joint and characterised the local surface morphology of the reconstructed joint surface at the most common defect sites, within a specific patient population eligible for FKRI treatment. Heat maps of the cartilage defect location revealed concentration of the defects on specific locations on both medial and lateral condyles. The local ROC were quantified for the 200 generated cartilage models for the locations that exhibited a high frequency of defect occurrence using the median defect size and aspect ratio for isolating a surface area. The proposed methodology in this study, which combines data on defect location and defect size with the local ROC measurements, provides detailed information that can be useful for designing treatments specifically tailored to address focal cartilage defects.

The heat maps, as used in this study, provide a clear and intuitive way of depicting the areas on the cartilage surface that are most affected by defects. Although not utilised in our study, artificial intelligence‐based automated MRI segmentation and annotation tools [20, 45] pave the road for clinical implementation of 3D MRI visualisation of cartilage injuries to aid in patient counselling and surgical care. 3D visualisation of predicted cartilage injury progression is a promising future prospect [38], which offers potential for patient counselling and could help incentivize compliance to conservative therapies such as weight loss therapy. In addition, 3D visualisation helps orthopedic surgeons plan and execute their surgical interventions to improve defect coverage and implant or graft congruency for techniques like FKRIs [25], osteochondral autografts [54] or allografts [39].

The use of SSMs can potentially support clinical decision making in the future if further research identifies that specific knee morphology variations are associated with response to treatment, as has been recently demonstrated for medial post meniscectomy syndrome patients [19]. Further research is warranted to elucidate what input variables need to be included in models to predict response to treatment in cartilage repair. Knee morphology variations, exact cartilage defect location, defect size and morphology, quantitative and morphological MRI parameters such as cartilage tissue thickness, meniscal integrity and biochemical composition, demographic variables, kinematic or kinetic gait parameters, leg mechanical axis, and a wide variety of blood or synovial fluid derived biomarkers are all potential candidate input variables for predictive models [12, 16, 17, 33, 53, 58]. Such prediction models could aid in clinical decision to help surgeons select the best treatment option for each patient, which is especially relevant for middle‐aged patients who could benefit greatly from a carefully weighted decision between biological cartilage repair, restorative techniques such as FKRI, or knee replacement.

The observations illustrated in the heat map provide insight into the aetiology of the focal cartilage defects, as the cartilage defect hot spots coincide visually with areas that exhibit local contact pressure peaks during the stance phase of gait and show greatest cartilage thickness [57]. Focal cartilage defects emanate from damage to its collagen matrix, which can occur due to either a single excessive high‐impact load or repetitive subcritical loads [44]. Understanding the underlying cause of a cartilage defect and assessing the overall condition of the joint are critical for treatment planning, especially since surgical treatment of underlying comorbidities [62] or treatment supplementation with orthobiologics [8] has gained substantial interest. The pattern of defect locations in traumatic cartilage defects could differ from those in degenerative defects, as these cartilage defects often occur as a result of atypical movements and are often accompanied by concomitant ligamentous or meniscal injuries. Therefore, it would be interesting to draw comparisons between different patient populations, for example middle‐aged versus younger athletic patients or osteochondritis dissecans patients. By comparing patients with and without ligamentous or meniscal injuries, the effects of concomitant injuries could also be studied. Much larger patient cohorts are needed for such comparisons, highlighting the importance of (inter)national registries.

Previous studies on FKRI treatment have reported average defect sizes ranging from 2.3 cm^2^ to 2.9 cm^2^ [13, 27, 36, 40]. The median defect sizes obtained in this study for the medial (2.8 cm^2^) and lateral (2.0 cm^2^) condyles are in accordance with the previously reported values, suggesting that the patient population in our study is representative of the typical patient population. The characterisation of the aspect ratio of the cartilage defect in this study has not been done previously, whereas this metric has substantial implications in the context of FKRI design.

The exclusion criteria employed for patient selection in this study, based on the WORMS score for bone attrition, exclude cases with Grade 2 and 3 bone shape deformations that are not amenable to focal resurfacing treatment. Although this approach reduces the number of eligible cases for this study, it ensures exclusion of cases with condyle surface shape flattening. The presence of such cases will otherwise be incorporated into the SSM, influencing the subsequent generation of 200 cartilage models and affecting the accurate characterisation of surface morphology. Possible gender‐related differences were not considered.

The ROC of the femoral condyles has been previously characterised in a wide variety of manners, dependent on the specific research question or envisioned application [21, 43]. Total knee replacements have been designed according to different principles, varying from single‐radius to dual‐radius and gradually‐reducing radius designs [41], and each design method requires input data acquired according in a corresponding manner. Comparison of the magnitudes of the ROC values obtained in this study to most of the values reported in literature is not relevant due to the methodological differences and the differences in envisioned application (TKR versus FKRI implant design). Du et al. [14] have characterised the anteroposterior ROC for osteochondral allograft matching purposes, which is quite similar to FKRI implant design in terms of envisioned application, as the ultimate clinical objective in both cases is joint resurfacing. Du et al. characterized the ROC over a much larger arc length in comparison to our study, and therefore attained different ROC magnitudes (31.9 mm/25.7 mm for MFC/LFC respectively, versus 40.8/33.8 mm in our study). However, the ratio of the MFC/LFC ROC is very similar for both studies (1.24 versus 1.21). With respect to the mediolateral curvature, Nuno et al. [43] determined the mediolateral femoral condyle curvature over the whole width of the condyle and reported an average of 22.9 mm for the medial condyle and 24.4 mm for the lateral condyle, at points with similar locations to Points 3 in our study. We found median mediolateral curvature values of 17.5 mm and 22.6 mm for Points 3 on the medial and lateral condyles respectively, which again shows notable differences in terms of ROC magnitudes, but similarities in terms of MFC/LFC ratios. Our findings show that analysing the morphology of the femoral condyles in a localised manner, with a region of interest corresponding to the median cartilage defect size, provides different ROC magnitudes in comparison to literature values that are typically used for TKR design purposes.

It remains unknown what the ideal size of the region of interest for local ROC determination and what the most optimal ROCs are for generic FKRI design. Perhaps determination of the ROC across a larger surface area is needed to assure congruency between a FKRI and the adjacent cartilage. Also, it remains unknown whether the median or the upper or lower interquartile ROC values should be used for FKRI design purposes. Implanting a FKRI with an implant ROC larger than the condyle ROC is prone to cause partial implant protrusion, which in turn can lead to degeneration of opposing native cartilage tissue [35]. It is imperative to perform in silico verification of FKRI design by returning to a patient data set, performing virtual surgeries, and subsequently analysing implant fit in the patient population. Such a study will elucidate whether or not a given design is suitable for treating a large part of the patient population.

Currently, the limited number of defects on the lateral condyle present in the study population results in a lower level of detail in depicting the spatial distribution of lateral condyle defects, and the sample size on the trochlear region is too small to render a meaningful heat map. The ratio of medial condyle to femoral condyle and trochlear defects in our patient population is in line with previous reports [42, 60, 61]. Based on this ratio, we estimate that a total population size of 250–500 patients is needed to attain similar sample numbers (approximately 50) for the lateral condyle and the trochlea as for the medial condyle. The current study is a single‐center study, and compilation of databases from multiple centres or national registries would be needed for larger sample sizes.

Limitations of this study include the fact that the SSM was generated based on patient MRI scans with manually reconstructed joint surfaces, rather than MRI data from healthy individuals. However, our methodology can also be considered a strength, as the SSM was developed with input data that is representative of the intended patient population. All segmentations and articular surface reconstructions were controlled by an experienced musculoskeletal radiologist, indicating that a diligent approach was followed. Furthermore, we were not able to report the exact angular location of the cartilage defects and therefore not able to draw a comparison to other reports in literature, such as Du et al. [14]. We were not able to determine the orientation of the mechanical axis of the femur since the MRI scans did not contain the proximal head of the femur, and therefore we were not able to accurately determine the cylindrical axis of the knee in a standardised way. Finally, the single‐center nature of this limited the sample size. As this study was explorative in nature, only descriptive statistics were reported [14].

## CONCLUSION

Heat maps that provide insight into the spatial distribution of knee cartilage defects and an SSM of the distal femoral articular cartilage together provide essential morphological information for the design and optimisation of focal cartilage defect treatments, such as FKRIs. Heat maps revealed that femoral condyle cartilage defects most commonly occur in areas that that experience highest contact stresses during the stance phase of gait. The data and methods developed in this study pave the way for the development of the next generation of cartilage repair treatments and diagnostic workflows, potentially improving surgical outcomes and enhancing patient quality of life.

## AUTHOR CONTRIBUTIONS

Majid Mohammad Sadeghi, Erkan Aşık, Pieter Emans, Frank Zijta, Nazli Tümer, Gabrielle Tuijthof, and Alex Roth made substantial contributions to the conception or design of the work; or the acquisition, analysis, or interpretation of data; or the creation of new software used in the work. Majid Mohammad Sadeghi, Gabrielle Tuijthof, and Alex Roth drafted the work or revised it critically for important intellectual content. Erkan Aşık, Pieter Emans, Frank Zijta, Nazli Tümer, Gabrielle Tuijthof, and Alex Roth approved the version to be published. Majid Mohammad Sadeghi and Alex Roth agree to be accountable for all aspects of the work in ensuring that questions related to the accuracy or integrity of any part of the work are appropriately investigated and resolved.

## CONFLICT OF INTEREST STATEMENT

Pieter Emans and Alex Roth are shareholders of Avalanche Medical BV, a commercial entity developing a focal knee resurfacing implant. Erkan Aşık is an employee of Avalanche Medical BV. Pieter Emans has a consultancy agreement with Episurf Medical AB.

## ETHICS STATEMENT

This retrospective study received ethical approval from the Maastricht UMC+ with the number METC 2023‐3691.

## Data Availability

Data available on request from the authors.
